# *Coptidis rhizoma* ameliorates type 2 diabetes mellitus-related metabolic dysfunction-associated steatohepatitis by downregulating the IL-17RA/NF-κB signaling pathway

**DOI:** 10.3389/fphar.2025.1667937

**Published:** 2025-09-29

**Authors:** Ning Yao, Xin Wang, Yajie Chen, Yunjuan Wu, Ying Su, Xinxin Su, Yajuan Geng, Xiaoning Liu, Limin Tian

**Affiliations:** ^1^ The First School of Clinical Medicine, Gansu University of Chinese Medicine, Lanzhou, Gansu, China; ^2^ College of Integrative Chinese and Western Medicine, Gansu University of Chinese Medicine, Lanzhou, Gansu, China; ^3^ School of Public Health, Lanzhou University, Lanzhou, Gansu, China; ^4^ Department of Endocrinology, Sichuan Provincial People’s Hospital, University of Electronic Science and Technology of China, Chengdu, Sichuan, China

**Keywords:** type 2 diabetes mellitus, metabolic dysfunction-associated steatohepatitis, *Coptidis rhizoma*, epiberberine, IL-17RA/NF-κB signaling pathway, traditional Chinese medicine

## Abstract

**Background:**

*Coptidis rhizoma*, a botanical drug derived from the dried rhizome of Coptis species (e.g., *Coptis chinensis*), is characterized by abundant natural sources, significant bioactivity, and high safety. It holds considerable potential for translational applications in metabolic diseases, particularly in ameliorating type 2 diabetes mellitus (T2DM)-related metabolic dysfunction-associated steatohepatitis (MASH). However, mechanistic studies on *Coptidis rhizoma* remain limited.

**Purpose:**

This study aimed to evaluate the therapeutic effects of *Coptidis rhizoma* on hepatic histological and functional damage, metabolic disorders, and insulin resistance in T2DM-related MASH and investigate its underlying mechanisms.

**Methods:**

Two-day-old male C57BL/6J mice were subcutaneously injected with streptozotocin (200 μg/20 μL per mouse). At 4 weeks of age, the mice were weaned and switched to a high-fat diet until week 9 to induce T2DM-related MASH. Starting from week 5, *Coptidis rhizoma* decoction was administered via oral gavage for four consecutive weeks to conduct *in vivo* studies. Additionally, hepatocytes were isolated from the model mice and exposed *in vitro* to epiberberine, the active metabolite of *Coptidis rhizoma*, for cellular-level investigations.

**Results:**

*Coptidis rhizoma* significantly attenuated hepatic inflammatory lesions, reduced the non-alcoholic fatty liver disease activity score, improved liver function, and alleviated glucose and lipid metabolism disorders and insulin resistance in a dose-dependent manner in T2DM-related MASH mice. At the transcriptional level, key components of the interleukin-17 receptor A (IL-17RA)/nuclear factor kappa B (NF-κB) signaling pathway were upregulated in the hepatocytes of T2DM-related MASH mice, and both *Coptidis rhizoma* and epiberberine downregulated their expressions. Furthermore, *Coptidis rhizoma* and epiberberine suppressed the secretion of pro-inflammatory cytokines associated with the IL-17RA/NF-κB pathway in hepatocytes.

**Conclusion:**

*Coptidis rhizoma* ameliorates pathological phenotypes in T2DM-related MASH by inhibiting the IL-17RA/NF-κB signaling pathway, and its active metabolite epiberberine is involved in mediating these protective effects.

## Introduction

Nutritional imbalance resulting from disordered eating behaviors is a major contributor to type 2 diabetes mellitus (T2DM) and T2DM-related metabolic dysfunction-associated steatotic liver disease (MASLD) (Mădălina et al., 2025; Tzeng and Lee, 2024). Within the spectrum of MASLD, metabolic dysfunction-associated steatohepatitis (MASH) represents a critical watershed stage associated with reversed or increased risks of progressive liver injury, including hepatic fibrosis, cirrhosis, and hepatocellular carcinoma (HCC). The coexistence of T2DM and MASH synergistically exacerbates disease progression and adverse clinical outcomes, making it a major challenge in metabolic and liver disease management (Gancheva et al., 2024; Targher et al., 2021; Younossi and Henry, 2024).

Despite its clinical significance, effective pharmacological therapies for T2DM-related MASH remain limited. Current treatment strategies primarily rely on long-term lifestyle management and symptomatic treatment as the pathogenesis of T2DM-related MASH is complex and multifactorial, involving metabolic dysregulation, chronic inflammation, and progressive fibrogenesis (Wei et al., 2024). Although resmetirom, the first FDA-approved therapeutic agent for MASH with hepatic fibrosis, has shown promise in clinical trials, its real-world efficacy in T2DM-related MASH may differ from results observed in a randomized controlled trial (RCT), highlighting an urgent need for more effective and targeted treatment strategies (Do et al., 2025; Lian et al., 2024).

The pathogenesis of T2DM-related MASH involves a complex interplay between metabolic dysfunction and immune dysregulation, leading to a pro-inflammatory and pro-fibrotic liver micro-environment (Parola and Pinzani, 2024). Among the key immunopathological features of T2DM-related MASH, the activation and infiltration of helper T lymphocytes 17 (Th17) in the liver, along with the overproduction of the potent pro-inflammatory factor interleukin 17A (IL-17A), are the most characteristic histopathological changes that play a pivotal role in driving hepatic inflammation, insulin resistance (IR), and fibrogenesis. These processes are considered central to the initiation and progression of T2DM-related MASH, making the interleukin-17 receptor A (IL-17RA) signaling pathway a promising therapeutic target (Abdel-Moneim et al., 2018; Huby and Gautier, 2022; Kartasheva-Ebertz et al., 2022; Samy et al., 2024; Zi et al., 2022).

Traditional Chinese medicine (TCM) has long been utilized for managing metabolic disorders and has gained increasing attention for its multi-component, multi-target therapeutic potential with relatively favorable safety profiles (Zhang et al., 2021). *Coptidis rhizoma*, a well-known botanical drug recorded in the *Pharmacopoeia of the People’s Republic of China*, has been widely used for its “clearing heat and drying damp, reducing fire, and detoxifying” properties in TCM theory, and it is commonly prescribed for diabetes and related complications. Modern pharmacology research studies have demonstrated that *Coptidis rhizoma* exerts hypoglycemic effects by enhancing glucose consumption and inhibiting α-glucosidase and α-amylase activities (Wang et al., 2023; Xiao et al., 2020; Xu et al., 2021). In our previous clinical study, *Coptidis rhizoma*, as the key component of the TCM formula *Xiaozhi Huaxian decoction*, significantly improved the liver function, reduced IR and the serum triglyceride (TG) level, and attenuated hepatic inflammation and fibrosis in patients with MASH (Wang and Yao, 2017).

Among its bioactive constituents, epiberberine (EPI), a key alkaloid derived from *Coptidis rhizoma*, has been reported to ameliorate hyperglycemia, hyperlipidemia, oxidative stress, and IR in T2DM models and exert protective effects against MASH induced by a methionine- and choline-deficient diet (Meng et al., 2018; Zhang et al., 2024; Zhou et al., 2023). However, the potential therapeutic effects of *Coptidis rhizoma* and its active metabolite EPI on T2DM-related MASH have not been systematically investigated yet.

In the present study, we established a mouse model of T2DM-related MASH to mimic the metabolic–immune dysregulated liver micro-environment. Using a combination of *in vivo* pharmacological approaches, immunological assays, and *in vitro* molecular biology techniques, we aimed to validate the therapeutic effects of *Coptidis rhizoma* and EPI on liver pathology, glucose/lipid metabolism, and inflammation and elucidate their underlying mechanisms, particularly focusing on the IL-17RA/nuclear factor kappa B (NF-κB) signaling pathway.

## Materials and methods

### Reagents

Streptozotocin (STZ, purity ≥98%), IL-17A (purity ≥98%), phosphate buffer saline (PBS), 3,3′-diaminobenzidine tetrahydrochloride (DAB), dimethyl sulfoxide (DMSO), and DMEM high-glucose complete medium were obtained from Sigma-Aldrich (St. Louis, MO, United States). EPI (purity ≥98%) was supplied by MedChemExpress (Monmouth Junction, NJ, United States). Pentobarbital was obtained from Weikeqi Biotechnology Co., Ltd. (Chengdu, China). Heat-inactivated fetal bovine serum (FBS) was obtained from Cytiva-HyClone Laboratories Inc. (Logan, United States). The high-fat diet (HFD) was obtained from Jiangsu Xietong Pharmaceutical Bio-engineering Co. Ltd. (Nanjing, China). Hematoxylin–eosin (H&E) stain was purchased from Solarbio Science and Technology Co., Ltd. (Beijing, China). Alanine aminotransferase (ALT), aspartate aminotransferase (AST), glutamyl transpeptidase (GGT), total bilirubin (T-Bil), TG, and glucose kits were purchased from Olympus (Tokyo, Japan). Insulin (INS), IL-6, IL-1β, IL-12p40, and tumor necrosis factor alpha (TNF-α) enzyme-linked immunosorbent assay (ELISA) kits were obtained from Abcam (Cambridge, United Kingdom). Primers were synthesized by Biomed (Beijing, China). Reverse transcription kits and quantitative real-time polymerase chain reaction (qPCR) kits were acquired from TaKaRa (Kyoto, Japan). Antibodies against IL-17RA (rabbit polyclonal), nuclear factor kappa B activator 1 (Act1, rabbit monoclonal), tumor necrosis factor receptor-associated factor 6 (TRAF6, rabbit monoclonal), NF-κB p65 (rabbit monoclonal), goat anti-rabbit IgG (polyclonal), and TRIzol were purchased from Invitrogen (Carlsbad, United States).

### Preparation of *Coptidis rhizoma* decoction

*Coptidis rhizoma* (the dried rhizome of *Coptis chinensis* Franch., *Coptis deltoidea C.Y.* Cheng et Hsiao, or *Coptis teeta* Wall., family Ranunculaceae) was obtained from the Affiliated Hospital of Gansu University of Chinese Medicine (Lanzhou, China). The crude drug, sourced from Sichuan, China (batch no.: D2405047; production data: 20240508), is a geo-authentic variety and was authenticated by the College of Pharmacy, Gansu University of Chinese Medicine.

We adopted the water decoction extraction method for decoction and concentration, which is one of the traditional methods for extracting herbs in TCM. According to previous research (Wang et al., 2023; Wu et al., 2024), dried *Coptidis rhizoma* (9.0 g) was powdered, passed through a 2.0-mm sieve, combined with 30 volumes (w/v) of distilled water and boiled for 30 min. The extraction was performed twice, and the combined filtrates were centrifuged at 4,000 *g* for 10 min at room temperature. The supernatant was concentrated under reduced pressure to a final volume of 150 mL, yielding a final concertation of 60 mg/mL. The *Coptidis rhizoma* decoction was aliquoted and stored at −20 °C until use.

In the clinical application of TCM in the treatment of T2DM using *Coptidis rhizoma*, the dosage of *Coptidis rhizoma* usually ranges from 9 g to 30 g, depending on the severity of the condition (Pang et al., 2016; Zhu et al., 2025). The dosage for mice (1.17 g/kg/d) was determined based on the “equivalent dose ratio table for conversion of human and animal body surface areas,” corresponding to a common clinical dose of 9 g/day for adults with T2DM.

### Animal model and drug interventions

**FIGURE 1:**
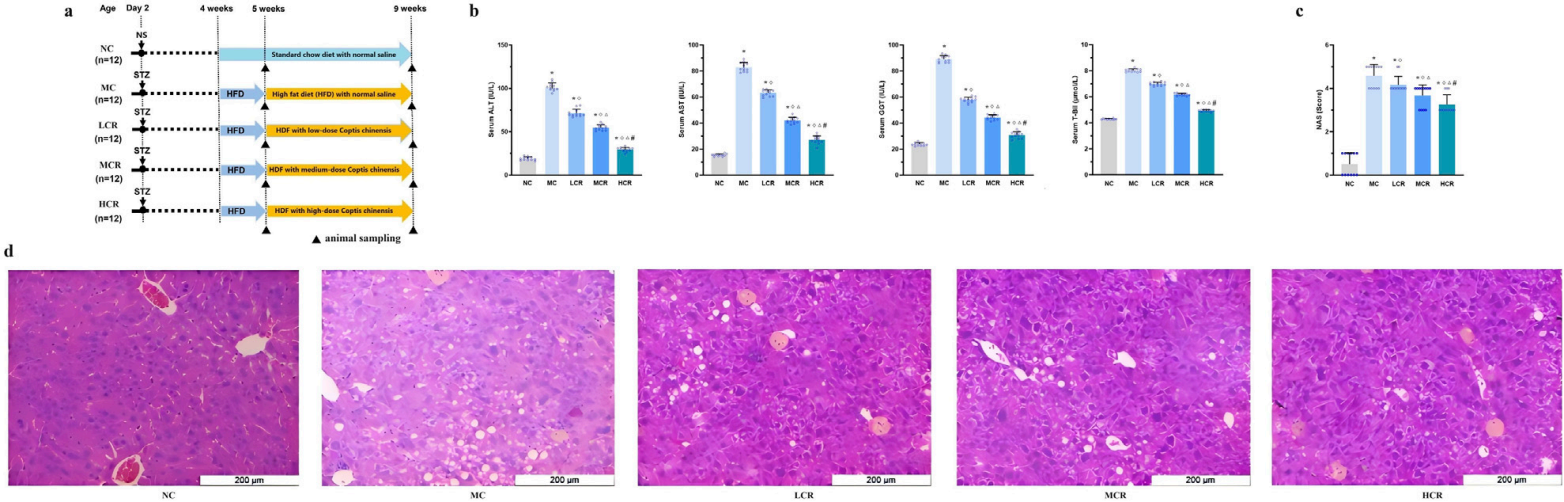
*Coptidis rhizoma* alleviated liver histological and functional lesions in T2DM-related MASH mice. **(a)** Schematic diagram of the experimental design. **(b)** Characteristics of liver functions. **(c)** Non-alcoholic fatty liver disease activity score. **(d)** Representative hematoxylin–eosin (H&E) staining of liver tissue. Data are presented as the mean ± SEM. *p < 0.001 vs. NC; ^◇^ *p* < 0.001 vs. MC; ^Δ^ *p* < 0.001 vs. LCR; ^#^ *p* < 0.001 vs. MCR. For Figure 1c, *p < 0.05 vs. NC; ^◇^ *p* < 0.05 vs. MC; ^Δ^ *p* < 0.05 vs. LCR; ^#^ *p* < 0.05 vs. MCR. Abbreviations for treatment groups: NC, normal control; MC, model control; LCR, low-dose *Coptidis rhizoma*; MCR, medium-dose *Coptidis rhizoma*; HCR, high-dose *Coptidis rhizoma*. Abbreviations for parameters: NS, 0.9% normal saline; STZ, streptozotocin; ALT, alanine aminotransferase; AST, aspartate aminotransferase; GGT, glutamyl transpeptidase; T-Bil, total bilirubin; NAS, non-alcoholic fatty liver disease activity score.

The experimental design is shown in Figure 1a. Sixty specific pathogen-free (SPF) male C57BL/6 J mice pups were obtained from the Experimental Animal Center of Lanzhou University (Lanzhou, China). According to previous research (Fujii et al., 2013), twelve mice were assigned to the normal control group (NC) and received subcutaneous injections of 0.9% normal saline (NS: 20 μL/head) at 2 days of age, followed by a normal chow diet after weaning at 4 weeks, and were maintained throughout the experimental period. The remaining 48 mice were injected with STZ (200 μg/20 μL/head, subcutaneously) at 2 days of age and fed an HFD after weaning at 4 weeks to induce the T2DM-related MASH model and maintained throughout the experimental period.

At 5 weeks of age, mice with fasting blood glucose (FBG) levels of >11.0 mM were considered diabetic and included in the study. These 48 mice were randomly divided into four groups (n = 12, per group): the model control group (MC), the low-dose *Coptidis rhizoma* treatment group (LCR, 0.585 g/kg/d), the medium-dose *Coptidis rhizoma* treatment group (MCR, 1.17 g/kg/d), and the high-dose *Coptidis rhizoma* treatment group (HCR, 2.34 g/kg/d). The NC and MC groups received equal volumes of distilled water. All treatments were administered by oral gavage twice daily for a duration of 4 weeks (i.e., until 9 weeks of age). All of the mice were housed under SPF conditions with a 12-h dark/light cycle and had free access to food and water during the experiment. All animal experiments were conducted strictly in accordance with the Laboratory Guidelines for Animal Experimentation. This study was approved by the Gansu University of Chinese Medicine’s Committee for Ethics of Animal Experimentation (approval no: 2021–036).

### Sample preparation

After the final gavage, mice were fasted for 12 h with free access to water. The mice were anesthetized via intraperitoneal injection of 3% pentobarbital. Blood was collected from the abdominal aorta and centrifuged to obtain the serum. Liver tissue was dissected from the same site to prepare the liver pathological section slices for histopathological analysis and liver homogenate.

### Primary hepatocytes preparation

Following Song et al. (2021), primary hepatocytes were isolated from mouse liver tissue using enzymatic digestion under sterile conditions at 4 °C. The cells were re-suspended in DMEM high-glucose medium supplemented with 10% (v/v) FBS, penicillin (100 U/mL), and streptomycin (100 μg/mL) and cultured in a Petri dish coated with type I rat tail collagen in a humidified incubator at 37 °C with 5% CO_2_. Cell viability was assessed by trypan blue exclusion (>90% viable).

### Cell incubation and IL-17A induction

IL-17A and EPI were dissolved in PBS and DMSO, respectively, and stored at 4 °C. After 12 h of culture, primary hepatocytes were harvested, and cell viability was confirmed by trypan blue exclusion (>90% viable).

Experiment 1 Primary hepatocytes from the NC, MC, LCR, MCR, and HCR groups were plated into new Petri dishes (1 × 10^5^ cells/dish) and treated with DMEM high-glucose medium containing 10% (v/v) FBS, penicillin, streptomycin, and IL-17A (the final concentration of 100 ng/mL) for 24 h (Peng et al., 2025; Shen et al., 2017). The cell-free supernatant was harvested for pro-inflammatory cytokine analysis.

Experiment 2 Primary hepatocytes from the MC group were divided into two groups: blank control (BC) and EPI pre-treatment (EP) with 1 × 10^5^ cells/dish. Cells were pre-treated with DMSO (BC) or EPI (100 μmol/L, EP) for 12 h, followed by stimulation with IL-17A (100 ng/mL) for another 24 h (Peng et al., 2025; Shen et al., 2017; Zhou et al., 2023). Both cell pellets and cell-free supernatants were harvested for mRNA expression and pro-inflammatory cytokine secretion analysis. Each experiment was repeated three times.

### RNA extraction and qPCR analysis

Total RNA was extracted using TRIzol reagent following the manufacturer’s instructions. The ratio of the absorbance at 260 nm and 280 nm (A260/280) was used to assess the purity of the RNA. The A260 was measured to determine the RNA concentration. cDNA was synthesized from 1 μg of total RNA using random primers. qPCR was performed using the Bio-Rad IQ5 system with SYBR Green Premix Ex Taq (Bio-Rad Laboratories, Inc., CA, United States), following the 2^−ΔΔCt^ method (Ross et al., 2003). The thermal cycling program was as follows: 95 °C for 5 min, followed by 35 cycles of 95 °C for 10 s, 55 °C for 20 s, and 72 °C for 20 s. Melting curve analysis was used to confirm amplification specificity. The primer sequences are listed in Table 1. Each sample was run in duplicate. β-Actin was used as the internal control.

**TABLE 1:** Primer sequences for qPCR.

Gene	Forward primer (5′ to 3′)	Reverse primer (5′ to 3′)
*IL-17RA*	GCTGAACACCAATGAGC	CCAAGGTCTCCACAGTG
*Act1*	AACGCAATAGATGGCTG	TCTCCAACTGCCTGATG
*TRAF6*	ATG​ACA​GCG​TGA​GTG​GCT​C	TCC​CTT​ATG​GAT​TTG​ATG​ATG​C
*NF-kB*	AAGCACTGTGAGGACGG	ATAGGCAAGGTCAGAATG
*β-actin*	GGGAAATCGTGCGTGAC	AGGCTGGAAAAGAGCCT

Abbreviations for parameters: IL-17RA, interleukin 17 receptor A; Act1, nuclear factor kappa B activator 1, TRAF6, tumor necrosis factor receptor-associated factor 6; NF-κB, nuclear factor kappa B.

### Immunohistochemistry analysis

The immunohistochemistry (IHC) analysis was performed as in the previous report (Shimaa et al., 2018). Liver tissue sections (5 μm) were deparaffinized, rehydrated, and subjected to antigen retrieval. Sections were incubated overnight at 4 °C with the following primary antibodies: rabbit polyclonal anti-IL-17RA (2.5 μg/mL), rabbit monoclonal anti-Act1 (2.0 μg/mL), rabbit monoclonal anti-TRAF6 (1.8 μg/mL), and rabbit monoclonal anti-NF-κB p65 (2.0 μg/mL). Rabbit serum replacing primary antibody was used for the incubation of the negative control samples. After washing, the sections were incubated with biotinylated goat anti-rabbit IgG, followed by peroxidase-conjugated streptavidin. DAB was used for color development, and sections were counterstained with hematoxylin. Brown–yellow staining indicated positive immunoreactivity. Finally, the sections were observed under a microscope, and the integrated optical density (IOD) value/area was quantified using Image-Pro Plus (v.6.0) software to analyze the differences in the protein expression levels.

### Histopathological analysis

The fixed liver tissue was dehydrated through an ethanol gradient, cleared with dimethylbenzene, embedded in paraffin, and cut into 4-μm-thick sections. H&E staining was performed according to the standard protocol. The non-alcoholic fatty liver disease (NAFLD) activity score (NAS) was calculated according to the criteria of Kleiner et al. (2005).

### Biochemical assays

The serum levels of ALT, AST, GGT, T-Bil, TG, FBG, and liver TG were measured by colorimetry using commercially available assay kits with the Olympus AU 640 Chemistry Analyzer (Shizuoka, Japan). All samples were run in triplicate.

### IR assessment

Serum INS was measured using ELISA kits following the manufacturer’s protocol with an ELx800 BioTek ELISA Reader at 450 nm (Vermont, United States). IR was determined by the homeostasis model assessment of IR (HOMA-IR) index according to the formula fasting INS (mIU/L) × FBG (mmol/L)/22.5, which has been previously validated against clamp measurements (Bonora et al., 2000).

### ELISA

The levels of IL-6, IL-1β, IL-12p40, and TNF-α in the liver homogenate and cell culture supernatants were measured using ELISA kits following the manufacturer’s protocol. The optical density was measured at 450 nm with an ELx800 BioTek ELISA Reader (Vermont, United States). All samples were run in triplicate.

### Statistical analysis

Data were analyzed using SPSS version 13.0 (Statistical Package for Social Science, Inc., Chicago, IL, United States). Continuous variables were summarized as the mean ± SD. The homogeneity of variance test and one-way ANOVA were performed. An LSD-t test was used to compare multiple groups if the variance was homogeneous and the null hypothesis was rejected, and a Tamhane’s T2 test was used to compare multiple groups if the variance was heterogeneous. All statistical analyses were based on two-sided hypothesis tests with a significance level of 0.05.

## Results

### *Coptidis rhizoma* alleviated liver functional and histological lesions in T2DM-related MASH mice

As shown in Figure 1b, T2DM-related MASH mice exhibited significant liver dysfunction, as evidenced by the markedly elevated serum levels of ALT, AST, GGT, and T-Bil compared with those of the NC group (all *p* < 0.001). Treatment with *Coptidis rhizoma* significantly reduced these hepatic injury markers in a dose-dependent manner (all *p* < 0.001), indicating a clear dose–effect relationship.

NAS is an indicator of histological severity. The mean of NAS in the MC group was consistent with the moderate stages of steatohepatitis. Data showed that NAS was significantly higher in the MC group than in the NC *and Coptidis rhizoma*-treated groups (all *p* < 0.05, Figure 1c). *Coptidis rhizoma* treatment markedly decreased NAS in a dose-dependent fashion (*p* < 0.05).

Histopathological analysis (Figure 1d) revealed that mice with T2DM-related MASH developed prominent hepatocellular edema, extensive lipid droplet accumulation, and inflammatory cell infiltration in the hepatic lobule, with no evident hepatic fibrosis observed in this study. *Coptidis rhizoma* treatment significantly ameliorated these pathological changes in a dose-dependent manner, with the HCR group showing the most pronounced improvement.

### *Coptidis rhizoma* improved glucose and lipid metabolism disturbances in T2DM-related MASH mice

**FIGURE 2:**
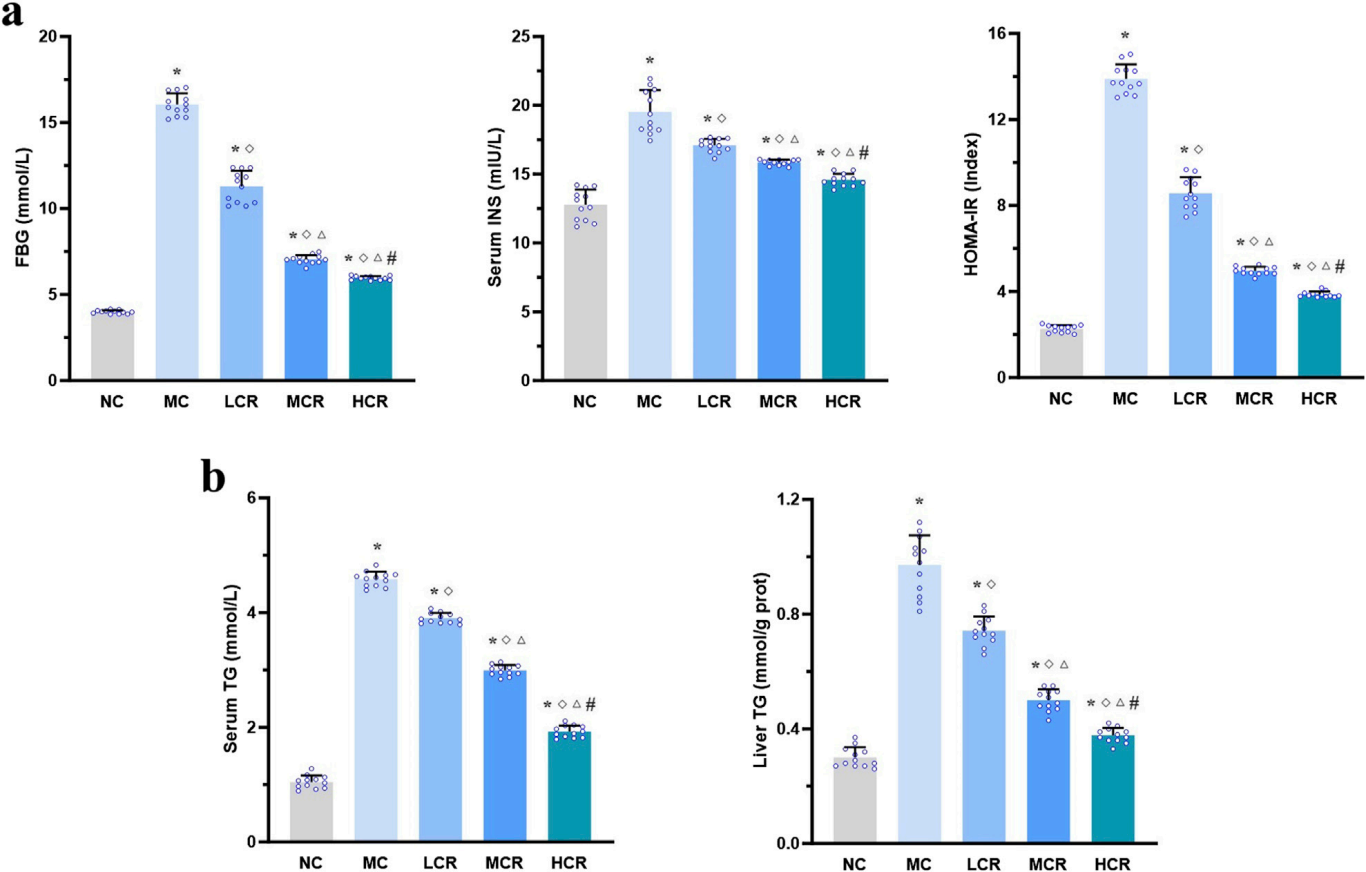
*Coptidis rhizoma* improved glucose and lipid metabolism disturbances in T2DM-related MASH mice. **(a)** Characteristics of glucose metabolism and insulin resistance. **(b)** Characteristics of lipid metabolism. Data are presented as the mean ± SEM. *p < 0.001 vs. NC; ^◇^ *p* < 0.001 vs. MC; ^Δ^ *p* < 0.001 vs. LCR; ^#^ *p* < 0.001 vs. MCR. Abbreviations for treatment groups: NC, normal control; MC, model control; LCR, low-dose *Coptidis rhizoma*; MCR, medium-dose *Coptidis rhizoma*; HCR, high-dose *Coptidis rhizoma*. Abbreviations for parameters: FBG, fasting blood glucose; INS, insulin; HOMA-IR, homeostasis model assessment of insulin resistance; TG, triglyceride.

As illustrated in Figures 2a, b, T2DM-related MASH mice displayed significant metabolic dysregulation, including elevated FBG, INS, HOMA-IR index, and serum and liver TG levels, compared with the NC group (all *p* < 0.001). *Coptidis rhizoma* treatment significantly improved these metabolic parameters in a dose-dependent manner (all *p* < 0.001), suggesting its beneficial role in restoring glucose and lipid homeostasis.

### *Coptidis rhizoma* reduced pro-inflammatory cytokine levels in the liver of T2DM-related MASH mice

**FIGURE 3:**
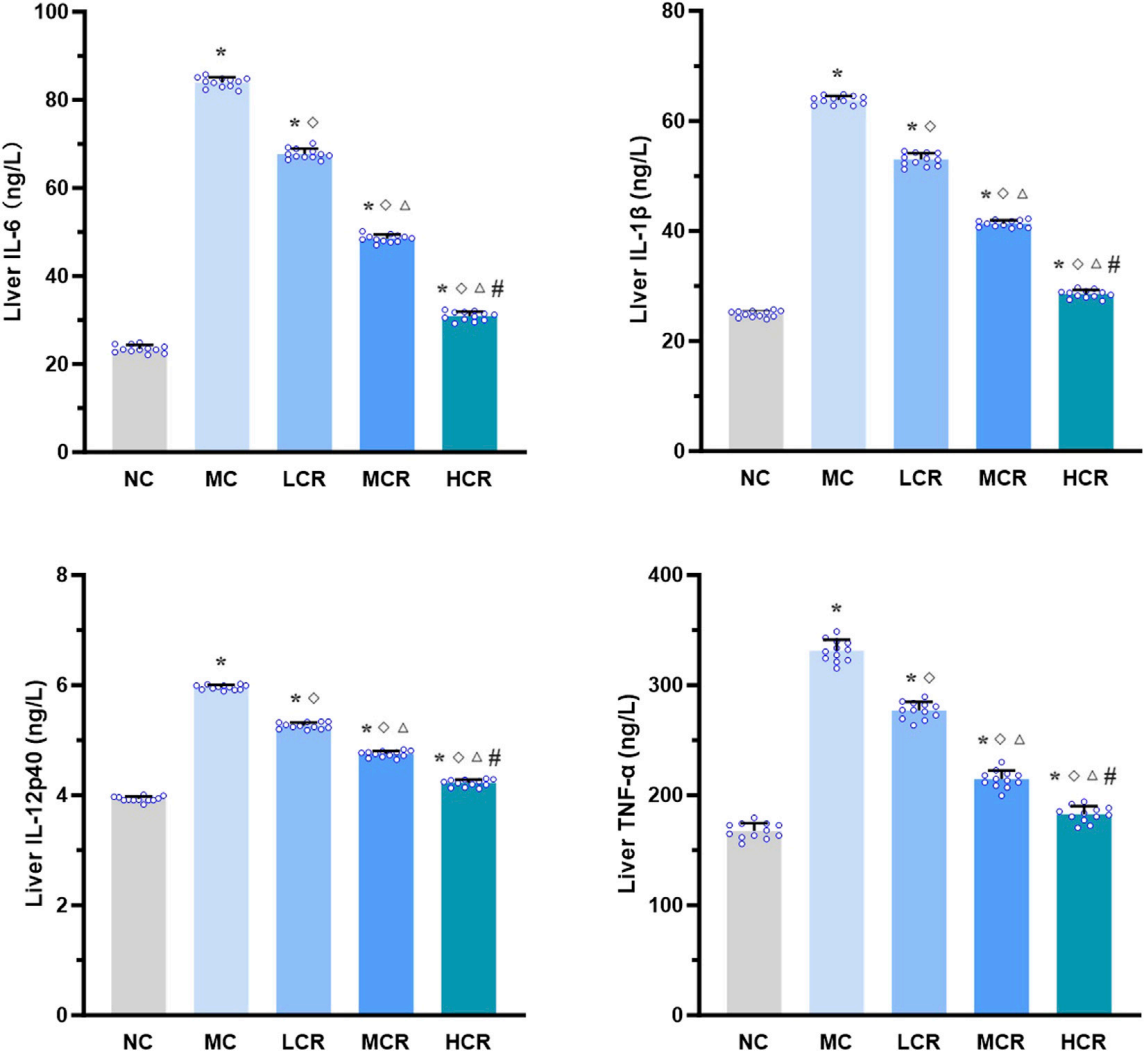
*Coptidis rhizoma* reduced pro-inflammatory cytokine levels in the liver of T2DM-related MASH mice. Data are presented as the mean ± SEM. *p < 0.001 vs. NC; ^◇^ *p* < 0.001 vs. MC; ^Δ^ *p* < 0.001 vs. LCR; ^#^ *p* < 0.001 vs. MCR. Abbreviations for treatment groups: NC, normal control; MC, model control; LCR, low-dose *Coptidis rhizoma*; MCR, medium-dose *Coptidis rhizoma*; HCR, high-dose *Coptidis rhizoma*. Abbreviations for parameters: IL-6, interleukin 6; IL-1β, interleukin 1 beta; IL-12p40, interleukin 12 P40; TNF-α, tumor necrosis factor alpha.

As shown in Figure 3, the hepatic levels of pro-inflammatory cytokines, including IL-6, IL-1β, IL-12p40, and TNF-α, were significantly higher in the MC group than in the NC group (all *p* < 0.001). Treatment with *Coptidis rhizoma* markedly decreased the levels of these cytokines (all *p* < 0.001). A clear dose-dependent reduction was also observed in the LCR, MCR, and HCR groups (all *p* < 0.001).

### *Coptidis rhizoma* downregulated the IL-17RA/NF-κB signaling pathway in the liver of T2DM-related MASH mice

**FIGURE 4:**
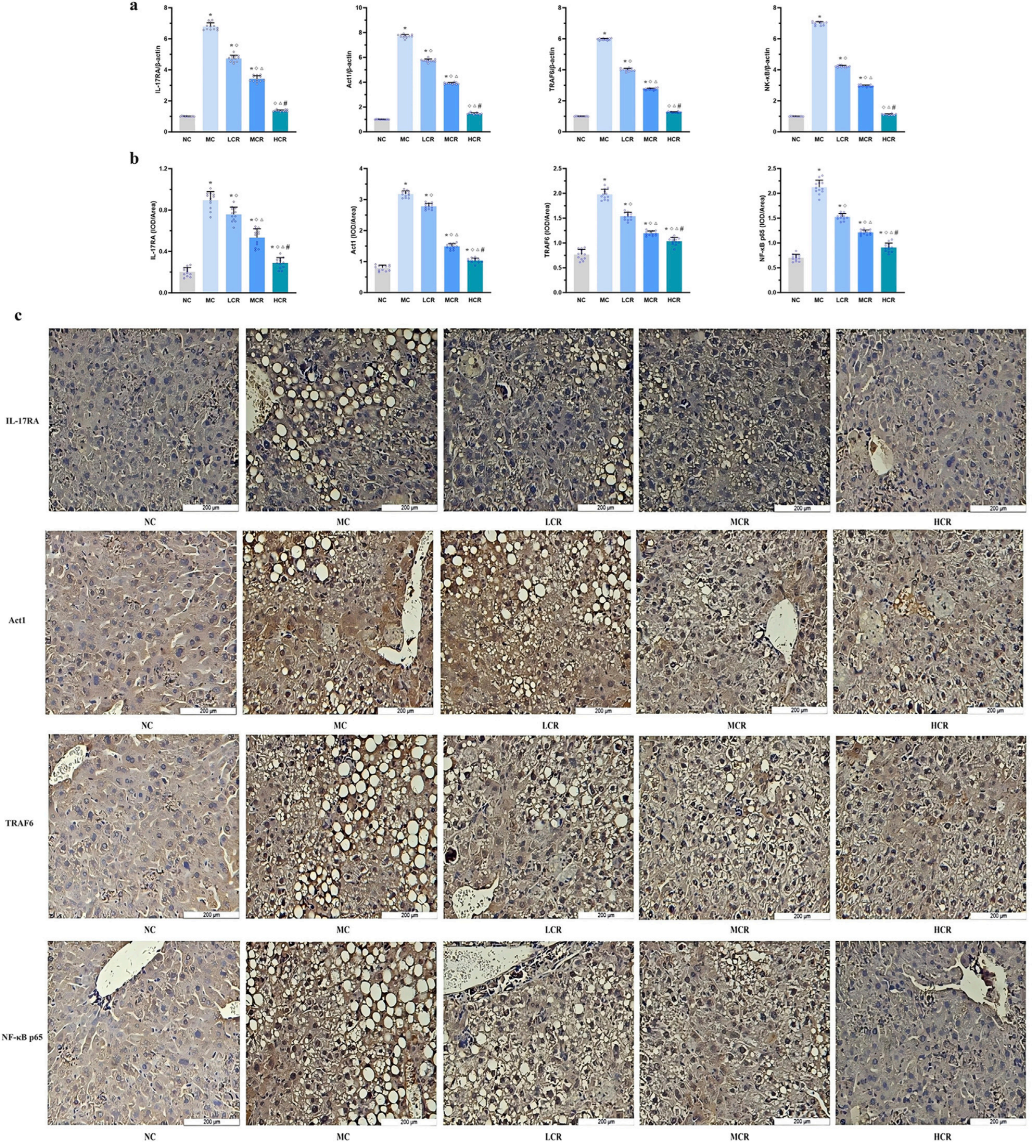
*Coptidis rhizoma* downregulated the IL-17RA/NF-κB signaling pathway in the liver of T2DM-related MASH mice. **(a)** mRNA expression of IL-17RA/NF-κB signaling pathway in the liver tissue. **(b)** Statistical graph using the integrated optical density value/area. **(c)** Representative images of IL-17RA/NF-κB signaling pathway expression detected by immunohistochemistry, in which positive staining was shown in brown. Data are presented as the mean ± SEM. *p < 0.001 vs. NC; ^◇^ *p* < 0.005 vs. MC; ^Δ^ *p* < 0.05 vs. LCR; ^#^ *p* < 0.05 vs. MCR. Abbreviations for treatment groups: NC, normal control; MC, model control; LCR, low-dose *Coptidis rhizoma*; MCR, medium-dose *Coptidis rhizoma*; HCR, high-dose *Coptidis rhizoma*. Abbreviations for parameters: IL-17RA, interleukin 17 receptor A; Act1, nuclear factor kappa B activator 1, TRAF6, tumor necrosis factor receptor-associated factor 6; NF-κB, nuclear factor kappa B; NF-κB p65, nuclear factor kappa B p65; IOD, integrated optical density.

As shown in Figure 4a, mRNA expression levels of the IL-17RA/NF-κB signaling pathway components were significantly upregulated in the MC group compared with those in the NC group (IL-17RA: approximately 6.79-fold; Act1: approximately 7.71-fold; TRAF6: approximately 5.96-fold; and NF-κB: approximately 6.99-fold; all *p* < 0.001). *Coptidis rhizoma* treatment significantly reduced the mRNA expression levels of IL-17RA, Act1, TRAF6, and NF-κB compared to those in the MC group (all *p* < 0.005), with a statistically significant dose-dependent effect among the treatment groups (*p* < 0.05).

IHC analysis (Figures 4b, c) further confirmed that the protein expression levels of IL-17RA, Act1, TRAF6, and NF-κB p65 were markedly increased in the MC group compared with those in the NC group. Treatment with *Coptidis rhizoma* downregulated the expression of these proteins in a dose-dependent manner, with the HCR group showing the most significant inhibition of IL-17RA/NF-κB pathway expression (all *p* < 0.001).

### *Coptidis rhizoma* inhibited IL-17A-induced pro-inflammatory cytokine secretions in the primary hepatocytes of T2DM-related MASH mice *in vitro*

**FIGURE 5:**
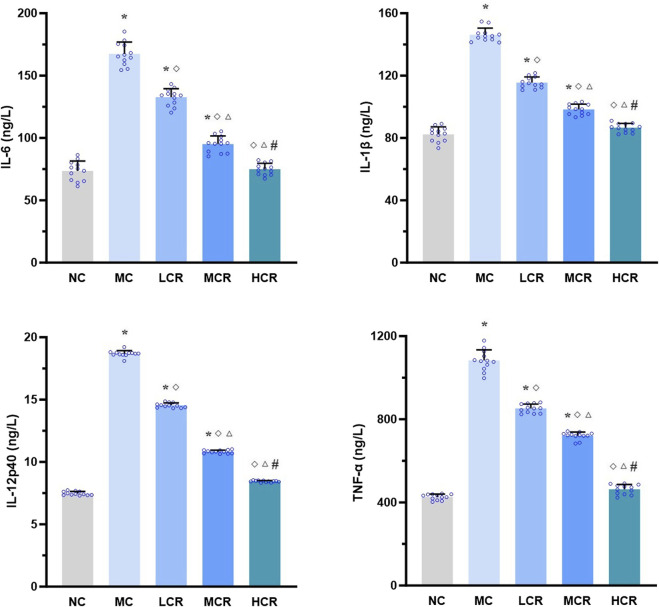
*Coptidis rhizoma* inhibited IL-17A-induced pro-inflammatory cytokine secretions in primary hepatocytes of T2DM-related MASH mice *in vitro*. Data are presented as the mean ± SEM. *p < 0.001 vs. NC; ^◇^ *p* < 0.001 vs. MC; ^Δ^ *p* < 0.005 vs. LCR; ^#^ *p* < 0.005 vs. MCR. Abbreviations for treatment groups: NC, normal control; MC, model control; LCR, low-dose *Coptidis rhizoma*; MCR, medium-dose *Coptidis rhizoma*; HCR, high-dose *Coptidis rhizoma*. Abbreviations for parameters: IL-6, interleukin 6; IL-1β, interleukin 1 beta; IL-12p40, interleukin 12p40; TNF-α, tumor necrosis factor alpha.

As shown in Figure 5, compared with that in the NC group, IL-17A stimulation significantly increased the secretion of IL-6, IL-1β, IL-12p40, and TNF-α by primary hepatocytes derived from T2DM-related MASH mice *in vitro* (all *p* < 0.001). Treatment with *Coptidis rhizoma* markedly suppressed the IL-17A-induced secretion of these cytokines (all *p* < 0.001), and the inhibitory effect was enhanced with increasing doses of *Coptidis rhizoma* (all *p* < 0.005).

### EPI downregulated the IL-17RA/NF-κB signaling pathway in the primary hepatocytes of T2DM-related MASH mice *in vitro*

**FIGURE 6:**
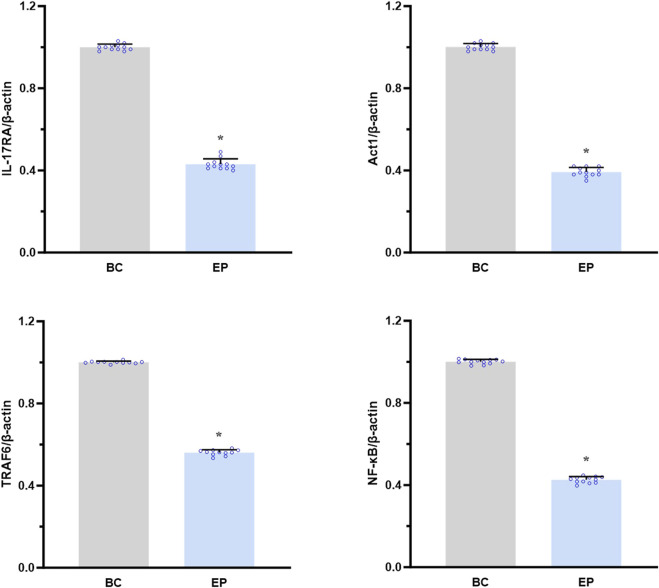
EPI downregulated the IL-17RA/NF-κB signaling pathway in primary hepatocytes of T2DM-related MASH mice *in vitro*. Data are presented as the mean ± SEM. *p < 0.05 vs. BC. Abbreviations for treatment groups: BC, blank control; EP, epiberberine pre-treatment. Abbreviations for parameters: IL-17RA, interleukin 17 receptor A; Act1, nuclear factor kappa B activator 1, TRAF6, tumor necrosis factor receptor-associated factor 6; NF-κB, nuclear factor kappa B.

As shown in Figure 6, EPI significantly reduced the mRNA expression levels of IL-17RA, Act1, TRAF6, and NF-κB in the primary hepatocytes from T2DM-related MASH mice compared to those in the BC group *in vitro* (IL-17RA: approximately 0.43-fold, *p* < 0.001; Act1: 0.39-fold, *p* = 0.001; TRAF6: 0.56-fold, *p* = 0.014; NF-κB: 0.42-fold, *p* = 0.002).

### EPI inhibited the IL-17A-induced activation of the IL-17RA/NF-κB signaling pathway in the primary hepatocytes of T2DM-related MASH mice *in vitro*

**FIGURE 7:**
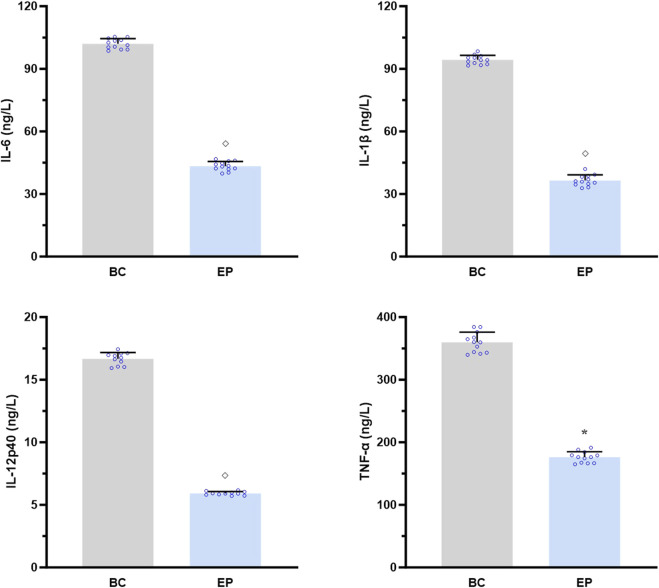
EPI inhibited the IL-17A-induced activation of IL-17RA/NF-κB signaling pathway in the primary hepatocytes of T2DM-related MASH mice *in vitro*. Data are presented as the mean ± SEM. *p < 0.05 vs. BC; ^◇^ *p* < 0.001 vs. BC. Abbreviations for treatment groups: BC, blank control; EP, epiberberine pre-treatment. Abbreviations for parameters: IL-6, interleukin 6; IL-1β, interleukin 1 beta; IL-12p40, interleukin 12p40; TNF-α, tumor necrosis factor alpha.

As shown in Figure 7, EPI dramatically suppressed the IL-17A-induced secretions of IL-6, IL-1β, IL-12p40, and TNF-α by the primary hepatocytes from T2DM-related MASH mice (*p* < 0.05 or *p* < 0.001), demonstrating its role in attenuating IL-17RA/NF-κB-mediated hepatic inflammation *in vitro*.

## Discussion

In recent years, T2DM-related MASH has emerged as a growing clinical concern due to its rising prevalence and potential for progressive liver damage, including hepatic fibrosis, cirrhosis, and HCC (Gancheva et al., 2024; Mădălina et al., 2025; Targher et al., 2021; Tzeng and Lee, 2024; Younossi and Henry, 2024). Despite its clinical significance, targeted and effective pharmacological therapeutic strategies for T2DM-related MASH remain limited in real-world settings, largely due to the complex interplay between metabolic dysregulation and immune-mediated liver injury (Do et al., 2025; Jain et al., 2025; Oladipupo et al., 2025).

Although resmetirom, the first FDA-approved therapeutic agent for MASH with hepatic fibrosis, has shown promise, its efficacy in patients with T2DM is not fully elucidated. Notably, a significant proportion of the participants in the pivotal phase III RCT exhibited well-controlled glycemia and limited diabetes-related complications (Harrison et al., 2024). In contrast, real-world T2DM populations often present with poor glycemic control, vascular comorbidities, and suboptimal treatment adherence. According to the American Diabetes Association, only 22.2% of T2DM adult patients have satisfactory control of glycemia, blood pressure, and plasma non-high-density lipoprotein cholesterol in the United States (American Diabetes Association Professional Practice C, 2024). Similarly, a national survey in China reported that merely 20.1% of adult T2DM patients attain optimal control of glycemia and blood pressure (Quan et al., 2023). Considering the differences between the real world and the RCT, the actual clinical effect of resmetirom on T2DM-related MASH may be biased against the RCT results and may not be fully generalizable (Lian et al., 2024). Therefore, there is an urgent need to develop safer and more effective treatment strategies that simultaneously address glucose and lipid metabolism dysfunction, IR, and hepatic inflammation in T2DM-related MASH.

Given the multifactorial pathogenesis of T2DM-related MASH, targeting a single pathway or molecular target is often insufficient to achieve comprehensive disease control. In contrast, multi-target, multi-level, and multi-angle therapeutic agents—especially those derived from natural products—can modulate various components of the disease network, thereby improving the therapeutic efficacy of T2DM-related MASH and reducing the adverse effects (Chen et al., 2021; Yuan et al., 2025).

*Coptidis rhizoma*, the dried rhizome of medicinal plants from the *Coptis* genus (e.g., *C. chinensis* Franch.) within the Ranunculaceae family, has been widely used in TCM for over a thousand years to treat “drinking and urine” syndrome, which is equivalent to DM in modern medicine and manifests as the main symptoms of “polydipsia, polyuria, polyphagia, emaciation, and fatigue” (Zhu et al., 2025). Phytochemically, over 120 bioactive metabolites have been isolated and identified from *Coptidis rhizoma*, including alkaloids, organic acids, lignans, flavones, and volatile oils, which collectively contribute to its multi-target pharmacological profile (Wang D et al., 2019).

In recent decades, accumulating evidence has demonstrated the potential of *Coptidis rhizoma* as a complementary and alternative therapy for T2DM and T2DM-related MASH. It has been shown to regulate FBG, improve lipid profiles, reduce IR, and mitigate lipid accumulation in hepatocytes, and it has a favorable safety profile (Cai et al., 2025; Gong et al., 2024; Jiang et al., 2025; Zheng et al., 2024; Zhu et al., 2025). Among its bioactive metabolites, EPI, a major alkaloid derived from *Coptidis rhizoma*, has been reported to alleviate IR by protecting T2DM from oxidative stress and suppressing hepatic TG synthesis to ameliorate liver steatosis by upregulating the small heterodimer partner and inhibiting the SREBP1/FASN pathway (Zhang et al., 2024; Zhou et al., 2023). However, most previous studies on *Coptidis rhizoma* have been conducted based on TCM compound formulas, making it difficult to clearly elucidate the effects and mechanisms of *Coptidis rhizoma* itself or its individual active metabolites on T2DM-related MASH.

A growing body of evidence has implicated IL-17A signaling in the pathogenesis of T2DM-related MASH, highlighting the IL-17RA/NF-κB axis as a promising therapeutic target (Elahi et al., 2024; He et al., 2021). In the present study, we established a mouse model of T2DM-related MASH that closely mimics the metabolic–immune-dysregulated liver microenvironment observed in clinical settings, thereby better reflecting the progression from simple steatosis to MASH under T2DM conditions (Fujii et al., 2013).

**FIGURE 8:**
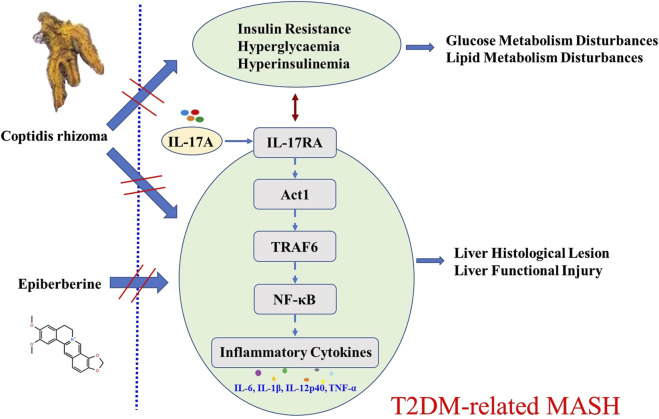
Schematic diagram for the mechanism of *Coptidis rhizoma* and EPI in ameliorating T2DM-related MASH. Abbreviations for parameters: IL-17A, interleukin 17 A; IL-17RA, interleukin 17 receptor A; Act1, nuclear factor kappa B activator 1, TRAF6, tumor necrosis factor receptor-associated factor 6; NF-κB, nuclear factor kappa B; IL-6, interleukin 6; IL-1β, interleukin 1 beta; IL-12p40, interleukin 12p40 TNF-α, tumor necrosis factor alpha.

Our findings reveal, for the first time, that *Coptidis rhizoma* effectively ameliorates T2DM-related MASH by downregulating the IL-17RA/NF-κB signaling pathway at both the overall and cellular levels. Upon binding of IL-17A (secreted by Th17 cells) to its receptor IL-17RA, downstream signaling through Act1, TRAF6, and NF-κB is activated, leading to the transcription of pro-inflammatory cytokines and exacerbation of hepatic inflammation and injury (Beringer and Moissec, 2018). We demonstrated that *Coptidis rhizoma* treatment significantly reduced the expression of IL-17RA, Act1, TRAF6, and NF-κB in a dose-dependent manner, thereby attenuating the inflammatory responses and improving liver pathology. The therapeutic effects were observed across three clinically relevant dosages of *Coptidis rhizoma*, corresponding to the typical human daily dosage range of 4.5 g–18 g for adult T2DM patients (Pang et al., 2016). Among the active metabolites, EPI played a pivotal role in mediating these effects. Our *in vitro* studies further confirmed that EPI inhibited IL-17A-induced secretion of pro-inflammatory cytokines and downregulated the expression of key IL-17RA/NF-κB pathway components in the primary hepatocytes. A schematic diagram illustrating the mechanism of *Coptidis rhizoma* and EPI in ameliorating T2DM-related MASH is shown in Figure 8.

TCM has long been utilized for managing metabolic diseases, with numerous botanical drugs showing promise in treating T2DM and its complications, including MASH (Yuan et al., 2025). Botanical drugs such as Fructus Corni, *Dracaena cochinchinensis*, *Panax ginseng*, mulberry, and oolong tea have demonstrated potential benefits in preclinical and clinical studies (Chen et al., 2017; Fan et al., 2014; Ng et al., 2018; Wang J et al., 2019; Wei et al., 2018). Although the diverse and complex chemical compositions of botanical drugs make it challenging to define precise molecular targets, their holistic effects have shown reliable therapeutic outcomes (Gong et al., 2024). *Coptidis rhizoma*, as one of the most widely used herbal components in TCM formulas for diabetes, holds significant potential as a safe and effective complementary or alternative therapy for T2DM-related MASH (Zheng et al., 2024). However, limited studies have focused on its use as a single botanical drug, highlighting an important gap in current research. Our study provides novel evidence supporting the therapeutic potential of *Coptidis rhizoma* and identifies EPI as a key bioactive component that mediates its anti-inflammatory and metabolic regulatory effects. These findings lay a solid foundation for the future development of evidence-based, single-herb, or component-based therapeutic strategies for T2DM-related MASH.

In the subsequent study, T2DM-related MASH mice will be injected with EPI (equivalent to the EPI dose in the *Coptidis rhizoma* extract) to assess its effects on liver function, metabolic indicators, and the pathway, directly validating EPI’s *in vivo* efficacy, along with identifying and scrutinizing the other specific active metabolites in *Coptidis rhizoma* to determine their individual contributions and potential synergistic effects in improving T2DM-related MASH. These subsequent studies will advance our understanding and enhance the potential and transformation of *Coptidis rhizoma* as a therapeutic strategy for T2DM-related MASH.

Despite its promising findings, this study has some limitations. We primarily focused on the role of the IL-17RA/NF-κB pathway and EPI in mediating the therapeutic effects of *Coptidis rhizoma*. Other potentially bioactive components of *Coptidis rhizoma*, such as berberine, may also contribute to its pharmacological effects through distinct or synergistic mechanisms, which are not yet elucidated (Wang D et al., 2019).

In future studies, we plan to directly evaluate the *in vivo* efficacy of EPI at doses equivalent to those delivered by the *Coptidis rhizoma* extract. We will also investigate other major active constituents of *Coptidis rhizoma* to determine their individual and combined contributions to the overall therapeutic effect. These efforts will further advance our understanding of *Coptidis rhizoma* as a translational therapeutic strategy for T2DM-related MASH and support its development into a clinically viable treatment option.

## Conclusion

This study demonstrates that *Coptidis rhizoma* ameliorates STZ- and HFD-induced T2DM-related MASH in mice. *Coptidis rhizoma* was shown to suppress the IL-17RA/NF-κB signaling pathway, promote liver histological and functional recovery, and improve glucose and lipid metabolism and IR, with the effects exhibiting a clear dose–response relationship. These therapeutic effects were closely associated with its active metabolite, EPI.

## Data Availability

The raw data supporting the conclusions of this article will be made available by the authors, without undue reservation.
